# Screening for texturing *Leuconostoc* and genomics behind polysaccharide production

**DOI:** 10.1093/femsle/fnaa179

**Published:** 2020-10-27

**Authors:** Vera Kuzina Poulsen, Anna Koza, Kosai Al-Nakeeb, Gunnar Oeregaard

**Affiliations:** Discovery, R&D, Chr. Hansen A/S, 10–12 Boege Allé, DK2970, Hoersholm, Denmark; Discovery, R&D, Chr. Hansen A/S, 10–12 Boege Allé, DK2970, Hoersholm, Denmark; Discovery, R&D, Chr. Hansen A/S, 10–12 Boege Allé, DK2970, Hoersholm, Denmark; Discovery, R&D, Chr. Hansen A/S, 10–12 Boege Allé, DK2970, Hoersholm, Denmark

**Keywords:** homo-polysaccharides, texture, screening, hetero-polysaccharides, wzy dependent pathway, *Leuconostoc*

## Abstract

Synthesis of polysaccharides by *Leuconostoc* can result in improved texture of fermented products. A total of 249 *Leuconostoc* strains were screened for homo-polysaccharide production and for texturing capabilities in milk. A total of six *Ln. mesenteroides* strains with superior texturing properties had the genetic blueprint for both homo- (HoPS) and hetero-polysaccharide (HePS) synthesis. Only one strain produced texture in milk without added sucrose, suggesting HePS synthesis via the Wzy dependent pathway. In milk acidification experiments with added sucrose, all six strains depleted the sucrose and released fructose. Thus, they can be used for both texture and possibly also for sweetness enhancement.

## Teaser

For the first time, a *Leuconostoc* strain was reported to give texture to milk by producing hetero-polysaccharides and not homo-polysaccharides.

## INTRODUCTION

*Leuconostoc* species are Gram-positive lactic acid bacteria (LAB) that can be found on vegetables, in silage and fermented food products such as cheese, quark, sour cream, kefir, sauerkraut and kimchi (Shin and Han 2015; Farkye 2017; Rezac *et al*. 2018). Taxonomic analysis indicates the close relationship of Leuconostocaceae and Lactobacillaceae, where Leuconostocaceae are placed as a monophyletic cluster within the Lactobacillaceae (Zheng *et al*. 2020). *Leuconostoc* strains grow poorly in milk on their own, due to lack of proteolytic activity. In dairy fermentations, *Leuconostoc* grow often in association with other mesophilic bacteria like *Lactococcus lactis* (Erkus *et al*. 2013).

Some *Leuconostoc* strains are known to produce HoPS such as dextran, mutan, inulin and alternan. HoPS are composed of only one type of monosaccharide, while HePS contain two or more types of sugars in their repeating units. *Leuconostoc* employ sucrase type enzymes to convert sucrose into HoPS consisting of either glucosyl units (glucans) or fructosyl units (fructans). The enzymes involved are glucansucrases (family GH70) and fructansucrases (family GH68), respectively, which typically use sucrose as the donor of the corresponding monosaccharide and transferring this residue to the growing HoPS and releasing the other monosaccharide to the environment (van Hijum *et al*. 2006). Exopolysaccharides bind water, and excessive production of HoPS in the presence of sucrose or raffinose leads to easily observable slimy colonies on agar surface. As raffinose is a substrate for fructansucrases but not for glucansucrases, MRS medium containing raffinose or sucrose can be used to distinguish if strains produce glucan or fructan (Malik *et al*. 2009). The sucrases are sucrose inducible, soluble or cell-wall bound enzymes that synthesize HoPS in the extracellular matrix (Bivolarski *et al*. 2013). This is different from the Wzy-dependent pathway for the production of HePS, which occurs intracellularly, and the repeating units are flipped to the outside the cell and polymerized into a growing HePS chain.

While the HoPS pathway has been described in the LAB genera *Weissella*, *Leuconostoc*, *Lactobacillus*, *Pediococcus* and *Streptococcus*, studies on Wzy-dependent pathway leading to HePS production focus almost exclusively on three genera: *Lactococcus*, *Lactobacillus* and *Streptococcus*. We have recently reported that gene clusters encoding the Wzy-dependent pathway are present in some *Leuconoctoc* strains (Zeidan *et al*. 2017). However, to the best of our knowledge, it has not been shown that *Leuconostoc* produce HePS.

Even though the potential to synthesize exocellular polysaccharides is encoded within a genome, it would not always lead to polysaccharide production. Also, polysaccharide production would not always lead to increased texture in a food matrix. The amount, the type and size of polysaccharides and their interaction with milk proteins are determining factors for texture development in milk (Hassan 2008; Mende, Rohm and Jaros 2016; Birch *et al*. 2017). Recently, we have reported a high-throughput texture screening method, based on milk fermentations in microtiter plates (MTP) combined with Total Aspirate Dispense Monitoring (TADM) pressure measurements (Poulsen, Derkx and Oregaard 2019).

The texture of fermented milk is an important quality parameter, affecting consumer acceptance. Finding *Leuconostoc* strains providing good texture can be beneficial for products such as sour cream and kefir. The aim of this work was to identify texturing *Leuconostoc* strains for use in fermented milk products and to investigate mechanisms behind the texturing properties.

## MATERIALS AND METHODS

### Strains

A total of 249 proprietary Chr Hansen strains identified by 16S rRNA sequences were used in this study (Table 1, Supporting Information). They were inoculated in 96 low-well MTP in 200 μL MRS-Difco broth and incubated overnight at 30°C and subsequently screened for HoPS (slimy colonies) or texture in fermented milk.

### High-throughput screening for slimy strains

A volume of 10 μL of overnight inoculum was transferred to MRS-Difco agar containing sucrose, raffinose or H_2_O (5%). After overnight incubation at 30°C, the slime production was scored by visual inspection of colonies.

### High-throughput screening for texturing strains

A volume of 20 μL of overnight culture was transferred to 1980 μL UHT skim milk (here called milk) containing pH indicator and 0.2% yeast extract, in 96 deep-well MTP and incubated at 24°C for 23 h when sucrose was added, or for 40 h in the absence of sucrose. The milk was prepared by reconstituting skim milk powder (Arla Foods, Denmark) to a level of dry matter of 9.5% and pasteurized at 99°C for 30 min, followed by cooling to 4°C. The ability of *Leuconostoc* strains to acidify milk was investigated using color-of-pH method and their texturing abilities were investigated using TADM as described in (Poulsen, Derkx and Oregaard 2019). TADM results (pressure versus time curves) were converted into single descriptors (TADM area) by accumulating all the measured pressure points below zero. The pressure was measured every 0.01 s for 3 s. Strains causing high texture in milk were represented by large TADM areas, whereas non texturing strains were represented by small TADM areas. We considered samples with TADM areas of above 800.000 as texturing, based on previous experience (Poulsen, Derkx and Oregaard 2019).

### Sugar measurements

The six texturing strains (0.9% inoculum) were used to co-ferment 200 mL milk containing 7 g/L sucrose and a *Lactococcus lactis* strain DSM 33133 (0.1% of inoculum) in baby bottles, where electrodes measured the pH development in real time. The milk acidifications were stopped at pH 4.55. Sucrose/D-Fructose/D-Glucose Assay Kit (K-SUFRG, Megazymes, Ireland) was used to measure sugar content in the fermented milk. To convert fermented milk samples (soft solids) into liquid samples, 333 µL of sterile TE-buffer (2 M Tris-HCl 0.2 M EDTA pH 8,0) was added to 1 mL fermented milk sample in a 2-mL MTP, incubated for 3 h at room temperature and centrifuged for 10 min at 2000 × *g*; 200 μL supernatant was transferred to a new MTP, several dilutions were prepared and tested by measuring OD changes following manufacturer protocol. The D-glucose concentration was determined before and after hydrolysis of sucrose by β-fructosidase (invertase). The D-fructose content was determined subsequent to the determination of D-glucose, after isomerization by phosphoglucose isomerase.

### Genome sequencing and assembly

The genomes of 137 strains were obtained by genomic DNA extractions, library preparation and quality control (QC) for *de novo* short read (Illumina, San Diego) whole genome sequencing (WGS). Moreover, the six texturing strains were additionally sequenced using long read sequencing by Oxford Nanopore Technologies, as the presence of transposases (IS elements) within the *eps* gene clusters made it difficult to assemble the large *eps* clusters in one contig. For species identification, BLAST analysis of the 16S sequences against the 16S rRNA-based LTP release 132 (https://www.arb-silva.de/projects/living-tree/) was performed based on the best-hit for each sequence. Genomic DNA for *de novo* short read WGS was extracted from 1 mL of overnight culture (at OD_600_∼1) with DNeasy Blood and Tissue kit on QiaCube system (Qiagen, Germany) following manufacturer protocol. Prior extraction, cell pellets were washed twice in TES buffer (50 mM TRIS pH 8.0, 1 mM EDTA pH 8.5 and 20% sucrose) and afterwards resuspended in 180 μL of pre-lysis TET buffer (20 mM TRIS-Cl pH 8.0, 2 mM EDTA pH 8.5, 1.2% Triton X-100, 20 g/L lysozyme, 2 µL 25 U/mL mutanolysin and 4 µL 100 g/L RNase A).

Genomic libraries were generated using modified Kapa Hyper Plus Library Preparation Kit (Roche, Switzerland) on Biomek i5 Liquid Handler (Beckman Coulter, Brea). 150 ng of genomic DNA (10 mg/L) diluted in 15 μL EB buffer (Tris-Cl, pH 8.0) was used in the half-volume reaction mixes for fragmentation, end-repair/A-tailing, ligation and final amplification. Conditioning solution (0.1 mM) was added to fragmentation mix and fragmentation time was optimized to 6 min. A total of 5 μL of 1 μM Kapa Dual-Indexed adapter (Roche, Switzerland) was used during adapter ligation step. A total of 10 μL of the adapter-modified DNA fragments were enriched by 8-cycle PCR. AMPure XP beads (Beckman Coulter) were used for two post-ligation and two post-amplification clean-ups (0.8×, 1.2×, 2 × 1×, respectively) to purify fragments at average size between 450 and 550 bp.

Concentration of gDNA and double stranded DNA libraries were measured by Qubit® 3.0 Fluorimeter using Qubit dsDNA Broad range and Qubit 1x dsDNA HS assays (Thermo Fisher Scientific, Waltham), respectively. Average dsDNA library size distribution was determined using the Agilent HS NGS Fragment (1–6000 bp) kit on the Agilent Fragment Analyzer (Agilent Technologies, Santa Clara). Libraries were normalized and pooled in the NPB (10 mM Tris-Cl, pH 8.0 and 0.05% Tween 20) to the final concentration of 10 nM. Denaturated in 0.2 N NaOH, 10 pM pool of libraries in 600 μL ice-cold HT1 buffer was loaded onto the flow cell provided in the MiSeq Reagent kit v3 (600 cycles) and sequenced on a MiSeq platform (Illumina Inc.) with a paired-end protocol and read lengths of 301 nt.

Genomic DNA extraction, library preparation and long read nanopore sequencing was performed by GenXone (Poland). Bacterial cell pellets were harvested by centrifugation (5 min, 6500 × *g*) from 5 mL overnight cultures (OD_600_ = 1.5). Genomic DNA was extracted by Genomic Maxi AX gravity column-based kit following manufactures guide (A&A Biotechnology, Poland). Purified DNA was then prepared for nanopore sequencing using the Rapid Barcoding Sequencing kit SQK-RBK004 (Oxford Nanopore, UK) following the manufacturer's protocol. The optional purification and concentration step with the Agencourt AMPure XP system (Beckman Coulter) was included into the procedure. Sequencing was performed on GridION X5 (Oxford Nanopore Technologies, UK) for 48 h. Genomic DNA and library concentrations were measured by Qubit 3.0 fluorometer and Qubit dsDNA High Sensitivity Kit (Thermo Fisher). Average fragment size distribution of DNA and libraries was obtained by using Fragment Analyzer and Genomic HS DNA 50 kb kit (Agilent).

Genome assemblies were made for all libraries using the tool Unicycler version 0.4.7 using a hybrid genome assembly approach. The raw Illumina short reads were trimmed using AdapterRemoval with the following non-default parameters: *‘–minquality 20 –minlength 30 –trimqualities –trimns –trim5p 15’*. The bacteriophage phiX is typically used in sequencing libraries as a control and the remnants of the phiX genome in the sequencing libraries may produce reads in the raw files, and must be removed before genome assembly. The trimmed reads were mapped to the complete genome of phi-X174 (NCBI accession id: J02482.1) using bwa mem (default parameters). The output of bwa mem was converted to SAM format by samtools using the following parameter: ‘*-f 12*’, where the -f 12 flag ensures that only unmapped paired reads are saved. The unmapped reads where converted back to the FASTQ-format using the SamToFastq function of the picard-tools suite (default parameters). The trimmed short reads and the raw ONT reads were then used as input to Unicycler with the following non-default parameter: ‘*conservative’* to produce a hybrid genome assembly.

### Mining for genes encoding polysaccharide biosynthesis

Mining for *eps* gene clusters was performed using BLAST analysis of the conserved part of the *Leuconostoc eps* gene clusters against the proprietary genomes. *Eps* gene cluster sequences were deposited in GenBank under the following accession numbers: Ln1, MT799691; Ln2, MT799688; Ln3, MT799689; Ln4, MT799690; Ln5, MT799687; Ln6, MT799692 and Ln7, MT799693.

Sucrases (GH70 and GH68) from 18 *Leuconostoc* strains publicly available in NCBI were used as references when mining for sucrase-like open reading frames (ORF) in the sequenced genomes.

Maximum likelihood phylogeny analysis (construction method: Neighbor Joining; Protein substitution model: WAG; Bootstrap analysis: 200) was performed using CLC Main Workbench 20. CAZyme annotations were performed using dbCAN meta server (http://bcb.unl.edu/dbCAN2/;Zhang *et al*. 2018). Clustal Omega (https://www.ebi.ac.uk/Tools/msa/clustalo/) using standard parameters was used for multiple sequence alignment and sequence identity calculation.

## RESULTS AND DISCUSSION

### Screening for HoPS production by *Leuconostoc* strains

The 249 *Leuconostoc* strains were screened for their ability to produce HoPS. They were drop plated on MRS agar supplemented with sucrose, raffinose or water. None of the colonies were slimy on MRS agar supplemented with water or raffinose, whereas 77 slimy colonies were observed with sucrose (31% of all the strains). These results indicate that the slimy strains produced glucan type HoPS. Most of the species had slime producing strains (Table 1, Supporting Information).

### High-throughput screening for milk texturing strains

Since exopolysaccharides are known to contribute to texture in fermented milk, the 249 strains were screened for texturing capabilities in milk. Since *Leuconostoc* strains have no proteolytic activity, the milk was supplemented with yeast extract, providing growth enhancing components including peptides and amino acids. The milk fermentations were conducted in MTP with and without 5% sucrose. When no sucrose was added, lactose, naturally present in milk, was the only carbon source. Not all strains were capable of acidifying milk.

**Figure 1.**
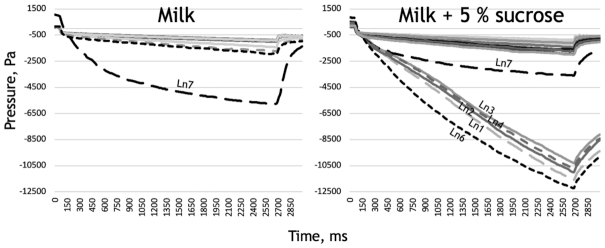
TADM curves of fermented milk samples. Strain Ln7 was observed to give high texture in milk (left panel). Strains Ln1, Ln2, Ln3, Ln4 Ln6 and Ln7 were observed to give high texture in milk supplemented with sucrose.

The texturing strains were identified by large TADM curve areas in fermented milk with and without sucrose. Figure 1 shows TADM curves of both selected representative non-texturing strains and the six identified texturing strains in milk supplemented with sucrose: Ln1, Ln2, Ln3, Ln4, Ln6 and Ln7, all belonging to the *Ln mesenteroides* species. A total of five of the texturing strains derive from fermented milk, while the origin of Ln7 is unknown (Table 2, Supporting Information).

### Texture as a result of HoPS and HePS production

**Figure 2.**
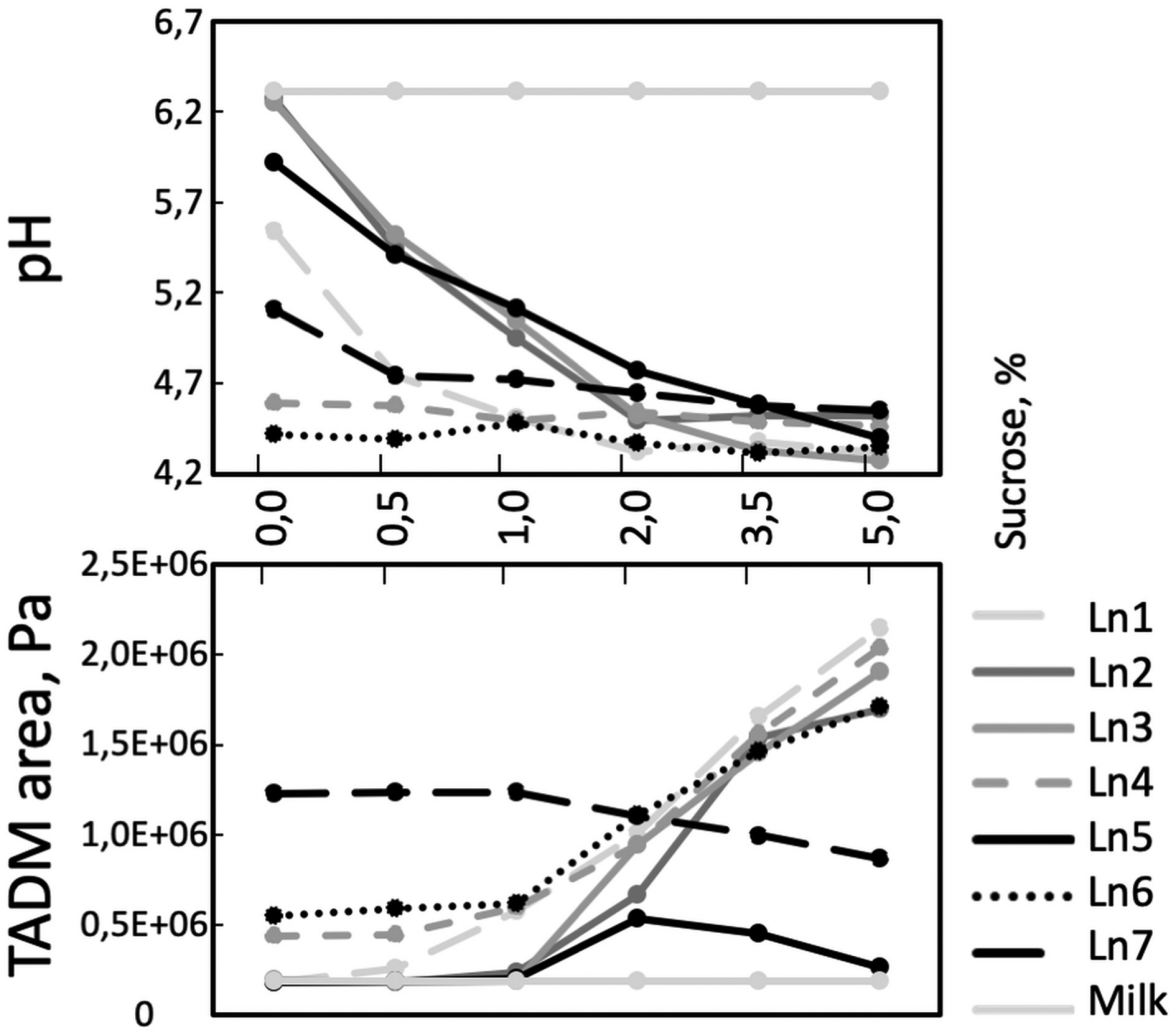
Influence of sucrose concentration on pH (top panel) and texture (bottom panel) of milk fermented with six texturing *Ln. mesenteroides* and a non-texturing HoPS producing control Ln5. The strains were incubated in milk containing yeast extract and different sucrose levels (0.0, 0.5, 1.0, 2.0, 3.5 or 5.0%) for 23 h at 24 °C. The values are means representing 4 to 24 biological replicates. For the pH measurements, the standard errors of the average values were between 0 and 0.1. For the TADM curve area measurements, standard errors of the average values were equal to or less than 5% for Ln1, Ln2, Ln3, Ln4, Ln5, Ln7 and non-inoculated milk and 8% for Ln6.

We hypothesized that the six texturing strains found in this work (Ln1, Ln2, Ln3, Ln4, Ln6 and Ln7) were producing HoPS from sucrose. In order to test this, we investigated the texturing capabilities of the six strains in both milk and in milk supplemented with various concentrations of sucrose. We also selected an additional non-texturing control strain, Ln5, that was observed to be slimy on MRS agar supplemented with sucrose, but not giving elevated texture in milk supplemented with 5% sucrose. The milk fermentations were done with yeast extract supplementation to allow for growth of *Leuconostoc*, and different sucrose levels (0, 0.5, 1.0, 2.0, 3.5 and 5.0%; Fig. 2). Two strains, Ln2 and Ln3, failed to acidify milk unless sucrose was present. Strains Ln1, Ln5 and Ln7 benefitted from sucrose addition, reaching lower end pH values with increasing sucrose levels. Only strains Ln4 and Ln6 had low end pH values independent on added sucrose. Ln5 had 2.8 and 2.4 times increase in TADM curve area in the presence of 2 or 3.5% sucrose, respectively, but with this relatively small TADM area it would not be considered a texturing strain.

It was observed that strain Ln7 had similar high TADM areas at 0, 0.5 and 1.0% sucrose; whereas there was a slight decrease in TADM area with further increasing sucrose levels (Fig. 2). The texture buildup in milk with no sucrose added indicates that the enhanced texture of Ln7 was the result of an active Wzy-dependent pathway, leading to HePS production and not from sucrase activity generating HoPS. To the best of our knowledge, Ln7 is the first described milk texturing *Leuconostoc* strain likely to be texturing independently of sucrase activity.

Strains Ln4 and Ln6 both had similar relatively low pH values independent of sucrose addition and both of these strains showed a clear dose dependent TADM area increase observable with sucrose levels 2.0, 3.5 and 5.0%. Strains Ln1, Ln2, Ln3 and Ln4 all showed substantial buildup of texture upon increasing levels of sucrose in the milk, observable at sucrose concentrations 2.0, 3.5 and 5.0%. These results suggest that the texture is dependent on the availability of sucrose and hence, likely due to buildup of glucan HoPS from sucrase activity.

### Genomics of polysaccharide biosynthesis in *Leuconostoc*

**Figure 3.**
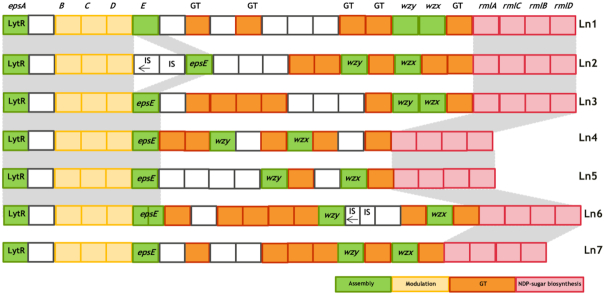
Schematic genetic organization of the *eps* gene clusters for the production of HePS in the six texturing *Ln. mesenteroides* strains and the non-texturing strain Ln5. Gene functional grouping is marked with different colors. Relative localizations of *eps* genes with homologous functions are indicated with connection bars. All the genes are transcribed in one direction except for a few genes oriented in the opposite transcriptional sense, which are indicated with arrows. Genes with unknown functions or functions not known to be related to the polysaccharide biosynthesis are in white; they include enzymes that might modify the oligosaccharide repeat unit structure, e.g. acyltransferases. Abbreviations: GT, glycosyltransferase; IS, transposase; NDP-sugar, nucleotide diphospho-sugar. Conserved regions between different strains of the *eps* gene clusters are marked with grey shadows. NDP-sugar biosynthesis genes abbreviated *rmlA*, *rmlC*, *rmlB* and *rmlD*, correspond to glucose-1-phosphate thymidylyltransferase or RfbA, dTDP-4-dehydrorhamnose 3,5-epimerase, dTDP-glucose 4,6-dehydratase and dTDP-4-dehydrorhamnose reductase, respectively. Genes in the *eps* operon were categorized into groups based on the putative or established functions of their products as in (Zeidan *et al*. 2017; Poulsen, Derkx and Oregaard 2019). These include modulatory genes (yellow; phosphoregulatory module *epsBCD*), polysaccharide assembly machinery genes (green; initiation *epsE*, polymerization *wzy*, export/flippase *wzx* and attachment *epsA/lytR*), genes encoding GT (orange; glucosyl transferases) necessary for the assembly of the repeating units, and genes encoding non-housekeeping functions (pink) required for the synthesis of activated sugar precursors and modification of the sugar residues.

We hypothesized that the observed texture in fermented milk was due to polysaccharide production, either by the Wzy-dependent pathway or due to sucrase activity. The genome sequences of the 137 *Leuconostoc* strains from the Chr. Hansen culture collection were mined for *eps* gene clusters and sucrase-like ORF. Genes associated with the Wzy-dependent pathway were observed in 14 out of 82 genome sequenced *Ln. mesenteroides*, including the six observed texturing strains (Fig. 3). The texturing strains also contained 2–5 sucrase-like ORF (Figure 1, Supporting Information). All sequenced *Leuconostoc* strains in this study had sucrase-like ORF encoding Glycoside Hydrolase Family 70 (GH70) enzymes. This suggests that these enzymes are important for the adaptation of *Leuconostoc* to its specific ecological niche (Yan *et al*. 2018b; Besrour-Aouam *et al*. 2019).

The overall structure of the *eps* gene clusters in the six texturing *Ln. mesenteroides* strains resembled that of the non-texturing strain Ln5 (Fig. 3) and other LAB (Zeidan *et al*. 2017). Genes located at the 5′ end of the *eps* gene cluster *epsABCDE*, which are involved in the modulation and assembly machinery of polysaccharide biosynthesis, displayed the highest level of conservation. Methionyl aminopeptidase (type I) like ORF was found in all the six strains between *epsA* and *epsB*; its role in the polysaccharide biosynthesis is unknown. The genes of the variable part including polymerase *wzy*, flippase *wzx* and glucosyltransferases (GT) or other polymer-modifying enzymes, were rarely similar between the strains, in agreement with what is observed in other organisms (Zeidan *et al*. 2017; Poulsen, Derkx and Oregaard 2019). At the 3′ end of the *eps* gene cluster, four conserved genes related to NDP-sugar biosynthesis genes were found in the six strains (Fig. 3).

In the near proximity of the *eps* gene clusters, several genes possibly related to the production of other cell wall polymers were found. A typical Gram-positive bacterial cell wall is composed of peptidoglycan and other cell wall polysaccharides such as wall teichoic acids, lipoteichoic acids and pellicles (Zeidan *et al*. 2017). Several putative domains for sugar binding, cell wall binding, wall teichoic acid and lipoteichoic acid synthesis, as well as synthesis of essential bacterial cell walls components such as peptidoglycan and galactofuranose, were found at the 3' end of the *eps* gene clusters in the six strains. Several IS elements were found within and surrounding the *eps* gene clusters.

Maximum likelihood phylogeny analysis revealed that *Ln. mesenteroides* and *Ln. pseudomesenteroides* contain very different sucrases (Figure 1, Supporting Information). All *Ln. pseudomesenteroides* except for one strain contained two dextransucrase-like ORF next to each other, as it is also the case in *Ln. pseudomesenteroides* AMBR10 available from NCBI (protein ID VTU60963.1, locus tag AMBR_MGDJBKAP_01842 and protein ID VTU60955.1 and locus tag AMBR_MGDJBKAP_01841) and they had 93–100% identity on the nucleotide level. Glucansucrase-like ORF from *Ln. mesenteroides* were much more diverse compared to those from *Ln. pseudomesenteroides*, and according to the phylogenetic analysis, were very different from those in *Ln. pseudomesenteroides*.

There are several groups of glucansucrases found in the strains in this study, based on the phylogenetic analysis (Figure 1, Supporting Information), which might correspond to the different subfamilies found within GH70 enzyme family (Gangoiti, Pijning and Dijkhuizen 2018; Yan *et al*. 2018a). Glucan products synthesized by family GH70 enzymes differ in size, type of linkages and degree of branching, leading to different physico-chemical properties. All the texturing strains contained two to five different sucrase-like ORF from the GH70 family. Interestingly, one sucrase-like sequence localized in the group dsr3a (Figure 1, Supporting Information) was found to be in common in all of the texturing strains, as well as Ln7 and *Ln. mesenteroides* strains LK-151 (protein ID BAX73443.1 and locus tag LEMES_02000) and DRC0211 (protein ID WP_014325090.1 and locus tag ARA01_RS07885). The presence of this 4581-bp long sequence with 99.13–99.98% identity on the nucleotide level between the six strains indicates that it might at least partially be responsible for the texturing properties of the strains.

One-third of the strains were slimy on agar containing sucrose, but only six strains were texturing in milk. Also, among 14 *Ln. mesenteroides* strains containing gene clusters for HePS production, only six were texturing in milk. This shows that the presence of genes encoding polysaccharide production *per se* does not indicate whether a strain will contribute with texture in a particular food matrix.

The future work would gain from the ability to isolate and quantify the mixture of different polysaccharides that are likely produced by the six texturing strains, in different conditions. It might be that methods like gel permeation chromatography or asymmetrical flow field-flow fractionation (AF4) combined with multi-angle laser light scattering (MALLS) would allow to separate the mixture of polysaccharides likely produced by these strains, and collect and quantify each polysaccharide separately. This would give a better understanding of the complexity of the exocellular polysaccharide production in *Leuconostoc*.

### Texture and sweetness with *Leuconostoc*

Sucrase activity will typically lead to buildup of glucans and release of fructose to the extracellular environment. In order to test if the texturing *Leuconostoc* strains could also contribute with sweetness to a fermented milk product, where it would usually be present in a mixture together with other strains, milk containing 7 g/L sucrose was fermented using a combination of *Leuconostoc* and a proteolytically active, milk fermenting *L. lactis* strain. In milk fermented with Ln1, Ln2, Ln3, Ln4 and Ln6, the fructose content was between 3.5 and 4.2 g/L, and that for Ln7 was 2.0 g/L, while the sucrose content was below the detection limit. These results indicate that the six texturing strains produce a glucose polymer and release fructose from sucrose into the environment. As fructose is 1.7 times sweeter and has a three times lower glycemic index compared to sucrose, the presence of 4.1 g/L fructose should taste as sweet as the presence of 7 g/L sucrose and result in a product with six times lower glycemic index. Unless the fructose released is metabolized and modified, the resulting product would be more slowly digested, absorbed and metabolized and would cause a lower and slower rise in blood sugar and, therefore usually, insulin levels. Thus, we believe that the texturing *Leuconostoc* strains producing HoPS and releasing fructose can be used to improve texture and retain sweetness.

## CONCLUSION

*Leuconostoc* have long been known for their ability to produce HoPS, which contribute to texture (Shin and Han 2015). We have previously observed that some *Leuconostoc* strains have the genes for HePS biosynthesis (Zeidan *et al*. 2017). In the present study, we found 14 *Ln. mesenteroides* strains that had the genes of the Wzy-dependent pathway. Interestingly, only six of these strains were texturing in milk supplemented with sucrose. Five out of six texturing strains showed increasing texture, when supplemented with sucrose (2.0–5.0%), suggesting dose dependent buildup of HoPS.

Ln7 was different from the other texturing strains, because it gave texture without sucrose, and with apparently fully functional *eps* gene cluster, we find it likely this strain secreted HePS into the extracellular matrix, causing texture buildup in the fermented milk. This is the first time that a *Leuconostoc* strain was reported texturing due to HePS production. This strain produced some HoPS, as indicated by the fructose release during milk acidification in the presence of sucrose, but the HoPS production did not apparently contribute positively to the texture.

## AUTHOR DETAILS

Chr. Hansen A/S, Bøge Allé 10–12, DK2970 Hørsholm, Denmark.

## Supplementary Material

fnaa179_Supplemental_Files
