# Supplementary material for: Screening for texturing Leuconostoc and genomics behind polysaccharide production
Source: FEMS Microbiol Lett. 2020 Oct 27;367(20):fnaa179. doi: 10.1093/femsle/fnaa179 (PMC7644082; doi:10.1093/femsle/fnaa179)
Supplement: fnaa179_Supplemental_Files [file fnaa179_supplemental_files.zip › 20201008_Supplem_Table1.docx]

### SUPPLEMENTARY DATA

**Supplementary Table 1.** Slime formation by *Leuconostoc* spp. used in this study. Slime formation was evaluated on MRS-Difco agar containing 5 % sucrose, 5 % raffinose or water as control, after an overnight incubation at 30 °C.

|  |  |  | **Number of tested strains with slimy colony appearance** | | |
| --- | --- | --- | --- | --- | --- |
| **Genus** | **species** | **Number of tested strains** | **H_2_O** | **Raffinose** | **Sucrose** |
| ***Leuconostoc*** | not determined | 8 | 0 | 0 | 2 |
| ***"*** | ***carnosum*** | 1 | 0 | 0 | 0 |
| ***"*** | ***citreum*** | 9 | 0 | 0 | 6 |
| ***"*** | ***fallax*** | 2 | 0 | 0 | 2 |
| ***"*** | ***lactis*** | 18 | 0 | 0 | 6 |
| ***"*** | ***mesenteroides*** | 149 | 0 | 0 | 31 |
| ***"*** | ***pseudomesenteroides*** | 62 | 0 | 0 | 30 |
|  | In total: | 249 |  |  | 77 |
