# Supplementary material for: Screening for texturing Leuconostoc and genomics behind polysaccharide production
Source: FEMS Microbiol Lett. 2020 Oct 27;367(20):fnaa179. doi: 10.1093/femsle/fnaa179 (PMC7644082; doi:10.1093/femsle/fnaa179)
Supplement: fnaa179_Supplemental_Files [file fnaa179_supplemental_files.zip › 20201008_Supplem_Table2.docx]

### SUPPLEMENTARY DATA

**Supplementary Table 2.** *Ln mesenteroides* strains containing HePS gene clusters and their characteristics: origin, growth in different carbon sources, slime and texture. Growth in different sugars was evaluated by incubating the strains overnight at 30 °C in 96 low-well microtiter plates in 200 μl MRS broth containing 2 % carbon source, as indicated. Slime formation was evaluated on MRS-Difco agar containing 5 % sucrose or raffinose. Texture in milk supplemented with 5 % sucrose was evaluated using TADM.
